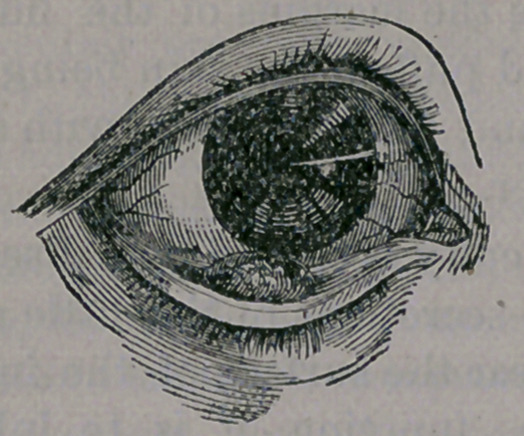# The Eye-Lids

**Published:** 1874-04

**Authors:** 


### THE EYE-LIDS.

The eye-lids are subjected to several varieties of disease; granulated lids, *entropium, trichiasis*, or inturning of the eye-lashes, all of which conditions have been fully treated upon in our former articles upon the eye. We now come to consider still another disease of the lid, known to oculists under the somewhat unintelligible name of *blepharitis ciliaris*, the English of which would mean, as nearly as we can translate the term, confluent pustules of the border of the lid. The affection is readily recognized, and doubtless has been seen by nearly all our readers, by reason of its great prevalence.

It is characterized by great redness and inflammation of the margins of the lids. The eye lashes are gummed together with a yellowish, sticky matter; which, when dry, scales off in a sort of drandruff, leaving the lids sore and bleeding. Often, the lashes are stuck together in the form of little brushes, the matter being hard, and adhering firmly to the lash and skin. Should it be forcibly removed, a sore and bleeding surface is presented beneath.

An apt similie, but a homely one, would be that, the eyes have the appearance of being held in place by circles of red putty.

The symptoms accompanying the disease, of course, vary in different individuals, and at various stages of its progression. Early in its development the extreme redness referred to, is very striking, giving a hideous appearance to the features, and causing great discomfort to the patient in the smarting and itching excited, and the feeling as though sticks or dust were lodged under the eye-lid. Later, the matter forming at the roots of the lashes and there encrusted, becomes so hard as to render it very difficult of removal. Matter forms under it, often accumulating to the dimensions of small abscesses, which rupture, discharging their contents, to further irritate the parts with which it comes in contact. In many cases the margins of the lids are much swollen and puffed, the skin being cracked or chapped, and fissures filled with a colorless, acrid matter.

The difficulty is caused by a diseased condition of the secretions of the little glands, distributed near the surface of the inside of the lid, whose function it is to lubricate the edges of the lids, for the purpose of preventing an overflow of the tears, as well as for anointing the eye and the lashes. These glands (the *meibomian*) when diseased, instead of discharging an oily substance, secrete an acrid and irritating matter, which in turn inflames the hair bulbs and the margins of the lids, in the manner described. A variety of causes exist for the little glands referred to becoming inflamed. Children or adults of a scrofulous habit are subject to it. Want of cleanliness, "straining the eyes," reading in smoke, or riding in the dust, etc., are some of the causes that produce it.

When the difficulty first makes its appearance, as is evinced in the matter forming at the roots of the lashes; wash the margins of the lids well, in warm water and castile soap. Remove all the matter, then rub some perfectly new and very salty butter among the roots of the lashes. Continue this treatment every night, until all trace of inflammation has subsided. In its worst form, go immediately to some skillful oculist nearest you, and place yourself under his care, otherwise it will soon produce an inflammation of the eye itself, which frequently results in complete destruction of vision. The disease is one that usually yields speedily and completely to the proper treatment, a few weeks only being necessary to complete a cure, in the most serious forms of the disease.

Still another difficulty of the lid is met with, having its origin in the same cause. When these little *meibomian* glands, referred to, become obstructed at their outlet, so that the oily secretion is not properly discharged, little tumors form in the lids, mostly in the upper ones, and are from the size of a grain of rice to that of a large sized pea. They are rarely painful, and scarcely would be noticed, were it not for their unsightly appearance.—Our illustration gives one of the lower lid, where they usually project more upon the inside of the lid, than is the case in the upper one.

They should be removed as early as possible as it can then be easily accomplished by simply opening the sac or tumor from the under side, allowing the contents to discharge—which will be found to be an amber colored oil—and the sac thoroughly cauterized. This procedure results in a speedy cure, but should only be undertaken by an experienced oculist, else might the cautery be allowed to come in contact with the *cornea*, and so produce a serious blemish. Such matters should only be entrusted to skillful men, and never to bunglers or traveling charlatans. As you would not likely entrust a valuable watch to a traveling tinker for repairs, neither should you employ a traveling doctor to prescribe for so valuable an organ as the eye.

## Figures and Tables

**Figure f1:**